# Application of the dual process model in end-of-life and bereavement interventions: a scoping review

**DOI:** 10.3389/fpubh.2026.1943201

**Published:** 2026-10-01

**Authors:** Qian Wu, Jinyun Zhang, Liangkun Xu, Xiaojun Ma, Bingxin Liu, Xiaoling Li

**Affiliations:** 1 School of Nursing, Shanxi Medical University, Taiyuan, China; 2 Shanxi Province Cancer Hospital / Shanxi Hospital, Chinese Academy of Medical Sciences, Affiliated Cancer Hospital of Shanxi Medical University, Taiyuan, China; 3 The First People's Hospital of Neijiang, Neijiang Affiliated Hospital of Chongqing Medical University, Neijiang, China

**Keywords:** bereavement, dual process model, end-of-life care, grief intervention, scoping review

## Abstract

**Objective:**

This study aims to review the current status of applications of the Dual Process Model (DPM) in end-of-life and bereavement interventions, and to provide a reference for the design of future DPM-based end-of-life and bereavement interventions and continuous support.

**Methods:**

Using the Joanna Briggs Institute (JBI) scoping review methodology and following the PRISMA-ScR reporting guidelines, we systematically searched Chinese and English databases for studies on the application of DPM in end-of-life and bereavement interventions. The included studies were charted and narratively synthesized.

**Results:**

A total of 17 studies were included. Existing research has primarily focused on bereavement adjustment after death, with a small number of studies addressing anticipatory grief among family members of terminally ill patients and continuous support before and after death. The scope of research has gradually expanded from individuals who have lost a spouse or partner to include those experiencing complicated or prolonged grief, family members of terminally ill patients, those who have lost loved ones to cancer, those who have lost loved ones in disasters or pandemics, and refugees, among other groups. DPM applications primarily fall into three areas: process identification and analysis, intervention structure design, and theoretical integration. Intervention content shows a dual emphasis on loss-oriented and restoration-oriented processes. Outcome measures tend to focus more on psychological indicators such as grief, depression, anxiety, and post-traumatic stress, while paying relatively less attention to process-oriented indicators emphasized by DPM, such as the oscillation process, life reconstruction, and dynamic regulation.

**Conclusion:**

DPM offers an important theoretical perspective for understanding the dual needs of coping with loss and resuming life in end-of-life care and bereavement. Existing studies indicate that DPM has been applied to various bereaved populations and intervention settings. However, its application during the end-of-life stage remains limited, and the oscillation process has not been sufficiently operationalized. Future research should further clarify how DPM guides intervention development, implementation adjustment, and outcome evaluation. Digital delivery and cultural adaptation should also be integrated to build continuous end-of-life and bereavement support across the transition before and after death.

**Systematic review registration:**

https://doi.org/10.17605/OSF.IO/HF7Q2.

## 1 Introduction

As the global population ages and the burden of chronic diseases such as malignant tumors increases, end-of-life care and bereavement support have become important psychosocial issues within public health systems (1–3). More than 60 million people die worldwide each year, and each death may have lasting effects on the mental health, family functioning, and social adjustment of multiple family members (4, 5). During the continuous process of adaptation from the end-of-life period through the bereavement period, family members experience anticipatory grief before death and continuing grief responses after the loss, while also facing practical challenges such as caregiving stress, role transitions, and the restructuring of their social lives (5–7).

During this process, most individuals gradually adapt to the loss over time, but some may experience persistent and severe grief reactions accompanied by significant impairment in social functioning (8). Surveys indicate that approximately 10% of bereaved individuals are at risk of developing prolonged grief disorder (PGD) and this risk may increase further in situations such as traumatic death, sudden public health emergencies, high caregiving burdens, or insufficient social support (9, 10) With the inclusion of PGD in the ICD-11 and DSM-5-TR, it is evident that pathological grief has become an important component of clinical services and public mental health interventions (11, 12). However, grief support should not be limited to the identification and treatment of pathological grief; it should also encompass continuous and tiered support for individuals in the end-of-life stage, those experiencing general bereavement, and high-risk populations (13). Existing intervention studies have largely focused on the expression of grief and the alleviation of psychological symptoms following a death, while paying relatively less attention to the stress of end-of-life care, the continuity of support before and after death, and the process of rebuilding one’s life after bereavement (14, 15). Therefore, end-of-life and bereavement interventions must not only focus on symptom relief but also address the dynamic process of adaptation as individuals navigate the transition from coping with loss to rebuilding their lives.

The Dual Process Model (DPM) proposed by Stroebe and Schut provides an important theoretical framework for understanding grief adaptation (16). This model posits that grief adaptation is not a linear process. Instead, it involves dynamic oscillation between loss-oriented (LO) and restoration-oriented (RO) processes (16, 17). LO processes involve dealing with the death event, the relationship with the deceased, and grief-related emotions; RO processes involve adapting to role changes, adjusting to daily life, and rebuilding life after loss. Previous theories of grief have largely explained the grief adaptation process from the perspectives of the work of grief, the stages of the process, and the tasks of mourning (18–20). In contrast, DPM is characterized by situating grief adaptation within a context where loss processing and life reconstruction coexist, emphasizing the individual’s constant transition between coping with loss and rebuilding their life (16). Based on this theoretical characteristic, DPM not only provides a dynamic perspective for explaining grief adaptation but also aligns with the situation faced by family members during the end-of-life stage, who are simultaneously confronted with anticipatory grief and the pressures of providing care, thereby providing a theoretical basis for developing continuous grief support before and after death (21).

In recent years, DPM has been used to understand experiences of bereavement, analyze coping processes, and study grief in specific contexts. Fiore noted that the DPM provides a clear explanation of the coping process among bereaved individuals and offers a theoretical basis for the design of grief interventions. However, the effectiveness of DPM-based interventions still requires further testing (22). Relevant empirical research indicates that DPM helps analyze changes in bereaved individuals’ coping strategies as they transition from grief processing to life recovery, and has received empirical support for the LO and RO processes; however, the oscillation process remains poorly understood (23, 24). Therefore, the application of DPM in grief support should not focus solely on the theoretical explanations but also further analyze how the theory is translated into concrete practices. This approach is also consistent with the view held in complex intervention studies that emphasizes the need to clarify the relationship among the theoretical basis, intervention components, mechanisms of action, and evaluation indicators (25).

A scope review helps identify the existing evidence and characteristics of conceptual application in a given research field; it is suitable for mapping the scope of application and modes of translation of DPM in research on end-of-life and bereavement support (26). Based on this, this study employed the Joanna Briggs Institute (JBI) systematic review methodology and followed the PRISMA-ScR reporting guidelines to systematically review the current status of DPM’s application in research on end-of-life and bereavement support (27, 28). This review focuses on target populations and contexts, ways in which DPM is applied, intervention content, and outcome evaluation characteristics. The findings may inform future research on DPM-based end-of-life and bereavement support and the development of intervention programs.

## 2 Methods

### 2.1 Study design

This study was conducted in accordance with the JBI scoping review methodological guidelines and was written in accordance with the PRISMA-ScR reporting guidelines. The research question was structured using the PCC framework: P (Population): individuals experiencing grief before or after the death of a significant person, including bereaved individuals, family members, and caregivers of patients at the end of life; C (Concept): the application of the DPM in end-of-life and bereavement support or intervention research; C (Context): end-of-life care, palliative care, hospice care, bereavement support, and other contexts involving grief before or after death.

### 2.2 Search strategy

Using a consistent search framework across both Chinese and English databases, we combined subject headings and free-text terms with Boolean operators (AND/OR/NOT). We systematically searched four Chinese databases (CNKI, Wanfang Database, VIP, and Sinomed) and six English databases (PubMed, Web of Science, Embase, Cochrane Library, CINAHL, and APA PsycINFO) from January 1, 1999, to May 21, 2026. Using PubMed as an example, Table 1 shows the search strategy and query.

**Table 1:** PubMed search strategy.

Steps	Search query
#1	“dual process model”[Title/Abstract] OR “dual process model”[Title/Abstract] OR “dual process model of coping with bereavement”[Title/Abstract] OR “dual process model of bereavement”[Title/Abstract] OR “DPM”[Title/Abstract] OR “loss-oriented”[Title/Abstract] OR “loss-oriented”[Title/Abstract] OR “restoration-oriented”[Title/Abstract] OR “restoration-oriented”[Title/Abstract] OR “oscillation”[Title/Abstract]
#2	“anticipatory grief”[Title/Abstract] OR “pre-loss grief”[Title/Abstract] OR “pre-death grief”[Title/Abstract] OR “predeath grief”[Title/Abstract] OR “anticipatory mourning”[Title/Abstract] OR “complicated grief”[Title/Abstract] OR “prolonged grief”[Title/Abstract] OR “traumatic grief”[Title/Abstract] OR “grief”[Title/Abstract] OR “mourning”[Title/Abstract] OR “mourn*”[Title/Abstract]
#3	“bereavement”[MeSH Terms] OR “bereaved”[Title/Abstract] OR “bereavements”[Title/Abstract] OR “widowhood”[Title/Abstract] OR “parental bereavement”[Title/Abstract] OR “spousal bereavement”[Title/Abstract] OR “ambiguous loss”[Title/Abstract] OR “ambiguous losses”[Title/Abstract]
#4	“terminal care”[MeSH Terms] OR “palliative care”[MeSH Terms] OR “hospice care*”[MeSH Terms] OR “hospice”[Title/Abstract] OR “hospice and palliative care nursing”[MeSH Terms] OR “end of life care*”[Title/Abstract] OR “terminal care”[Title/Abstract] OR “palliative*”[Title/Abstract] OR “hospice nursing*”[Title/Abstract] OR “palliative nursing”[Title/Abstract]
#5	#2 OR #3 OR #4
#6	#1 AND #5

### 2.3 Research questions

The research questions were identified through a review and analysis of the literature: (1) What are the primary target populations, contexts, and forms of support in DPM-based end-of-life and bereavement intervention studies? (2) How has DPM been applied in the design of end-of-life and bereavement support or interventions in relevant studies? (3) What evaluation measures have been used in DPM-related intervention studies, and do they reflect the dynamic adaptation process emphasized by DPM?

### 2.4 Inclusion and exclusion criteria

Inclusion Criteria: (1) The study participants are individuals experiencing end-of-life grief, anticipatory grief, or bereavement, as well as their family members or caregivers; (2) The research explicitly uses the DPM as a theoretical framework, basis for intervention, or analytical framework; (3) The research contexts include end-of-life care, palliative care, bereavement following death, or continuous care before and after death; (4) The study type is original research, including quantitative, qualitative, or mixed-methods studies; (5) The language of the literature is Chinese or English.

Exclusion criteria: (1) Studies not related to death-related loss; (2) Studies that did not explicitly use DPM; (3) Non-original or non-empirical publications, such as reviews, guidelines, commentaries, editorials, letters, study protocols, and conference abstracts; (4) Literature for which the full text was unavailable or that had been duplicated.

### 2.5 Literature screening and data extraction

We imported the retrieved literature into EndNote X9 and removed duplicate entries. Two trained researchers (WQ and ZJY) independently reviewed the titles and abstracts for the initial screening, applying the inclusion and exclusion criteria. They then reviewed the full texts for secondary screening. Inter-rater reliability between the two reviewers was assessed using Cohen’s kappa coefficient during both the title/abstract screening and full-text screening processes. If the researchers disagreed during screening or on the results, they discussed the issue or referred it to a third researcher (XLK) for a decision. Two researchers independently extracted information from the studies and cross-checked each other’s findings. The extracted information includes authors, study population, sample size, study setting, type of intervention, duration of intervention, use of DPM, intervention content, and outcome measures.

## 3 Results

### 3.1 Literature search results

A total of 1,150 records were identified from Chinese and English databases: China National Knowledge Infrastructure (CNKI; *n* = 9), VIP (*n* = 4), Wanfang Database (*n* = 54), China Biomedical Literature Database (CBM; *n* = 2), PubMed (*n* = 121), Embase (*n* = 149), Cochrane Library (*n* = 108), Web of Science (*n* = 399), CINAHL (*n* = 103), and APA PsycINFO (*n* = 201). After duplicate records were removed (*n* = 529), records remained for title and abstract screening (*n* = 621). After title and abstract screening, we excluded records (*n* = 554) and retrieved reports for full-text assessment (*n* = 67). After full-text review, we excluded reports (*n* = 52), leaving studies that met the inclusion criteria (*n* = 15). We identified additional studies through reference tracing (*n* = 2). Finally, we included 17 studies in this review (*n* = 17) (29–45). The inter-rater agreement between the two reviewers was substantial during the title and abstract screening (Cohen’s κ = 0.82) and full-text screening (Cohen’s κ = 0.74). Figure 1 shows the literature screening process.

**Figure 1:**
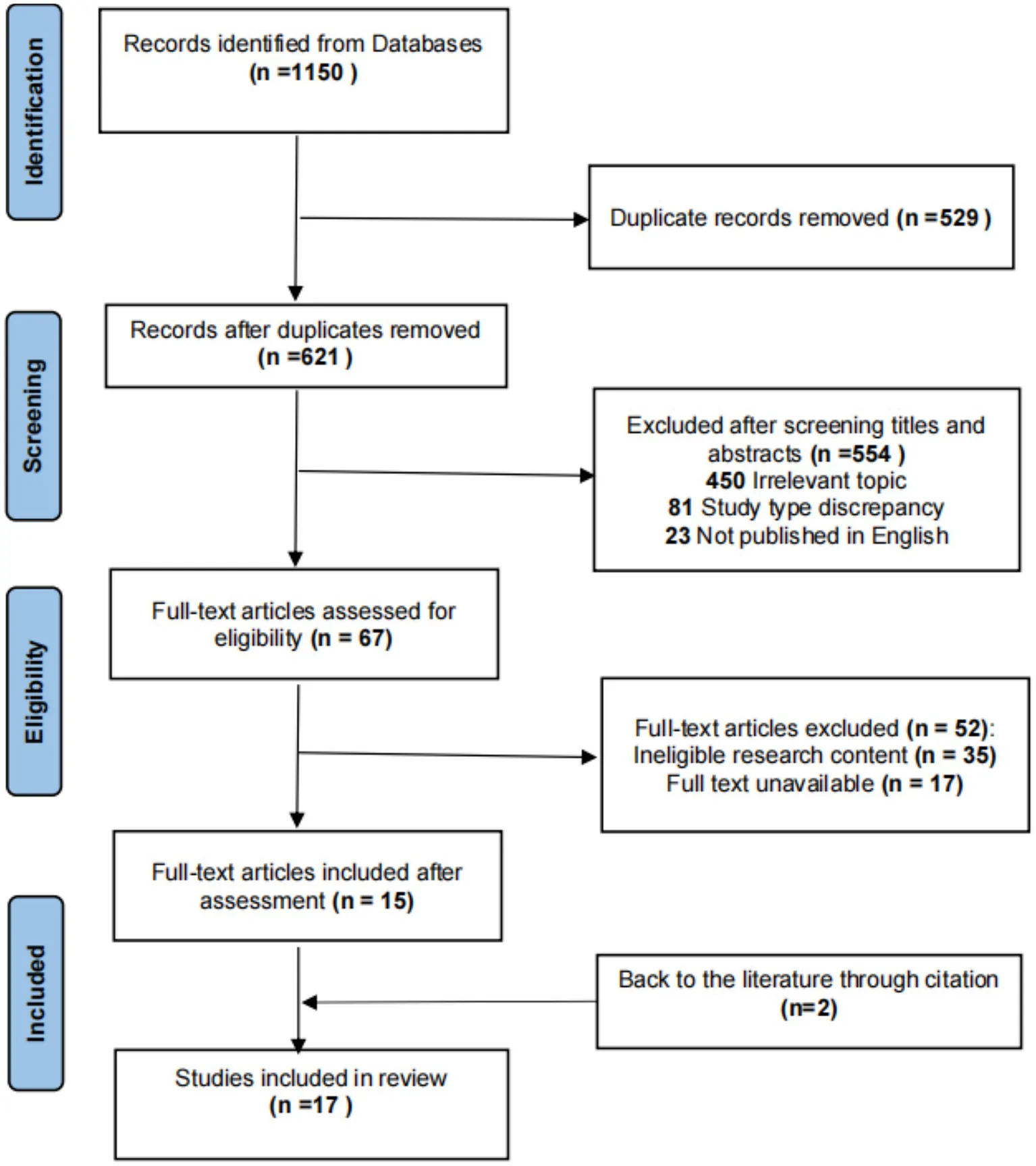
Flowchart of the literature screening process.

### 3.2 Basic characteristics of the included studies

This study included 17 studies published between 2010 and 2026. In terms of geographic distribution, the studies were concentrated in the United States (*n* = 8) (29–34, 38, 42) and China (*n* = 5) (36, 39, 41, 43, 45), with the remainder located in Australia (37), Canada (35), Switzerland (40), and Jordan (44). In terms of study design, most included studies were randomized controlled trials (RCTs) (*n* = 10) (29–32, 35, 36, 40, 42–44). The remaining studies included quasi-experimental studies (*n* = 3) (38, 39, 45), mixed-methods studies (*n* = 3) (33, 37, 41), and one descriptive study (*n* = 1) (34). Sample sizes ranged from 16 participants to 328 participants. The basic characteristics of the included studies are shown in Table 2.

**Table 2:** Basic characteristics of the included studies (*n* = 17).

Author/year	Study population	Sample size	Research context	Forms of intervention	Intervention time	DPM applications	Intervention content	Outcome measures
Lund et al. (29)	People aged ≥50 years who had lost a spouse or partner	298	2–6 months after the death of a spouse or partner	Group Psychosocial Intervention	14 weeks	Developing Living After Loss (LAL) interventions	Emotional expression and coping with Loss; Adjustment to life and role reconstruction; Modularity and dynamic organization	Approaches, course learning and usage, satisfaction
Utz et al. (30)	People aged ≥50 years who had lost a spouse or partner	328	2–16 months after the death of a spouse or domestic partner	Group Psychosocial Intervention	14 weeks	Designing content for the LAL Support Group	Emotional expression and coping with Loss; Adjustment to life and role reconstruction; Modularity and dynamic organization	Growth, self-care, and daily living skills related to sadness, depression, loneliness, and stress
Caserta et al. (31)	People aged ≥50 years who had lost a spouse or partner	328	2–18 months after the death of a spouse or domestic partner	Group Psychosocial Intervention	14 weeks	Proposing a hypothesis regarding LAL intervention and evaluating its effects	Emotional expression and coping with loss; Adjustment to life and role reconstruction; Modularity and dynamic organization	Coping strategies; coping balance; coping awareness;
Shear et al. (32)	Individuals aged ≥50 experiencing complicated grief	151	Loss of a spouse/partner, parent, child, or other significant other, and an ICG score ≥30	Individual psychotherapy	16 treatment sessions	DPM as one of the theoretical references for complicated grief treatment (CGT)	Emotional expression and coping with loss; Meaning reconstruction and sustained connection	Treatment response rate; severity and symptoms of complicated grief; loss-related avoidance; depression
Wittenberg-Lyles et al. (33)	Hospice caregivers after the loss of a loved one	16	Grief support for caregivers following the death of a hospice patient	Digital self-help intervention	9 months	DPM as a theoretical and analytical framework for the “Secret Facebook Group”	Emotional expression and coping with loss	Anxiety, depression
Caserta et al. (34)	Spouses or partners caring for cancer patients in hospice care	Not reported	Support for cancer caregivers: From end-of-life care through the bereavement period	Personalized grief support	14 weeks	DPM is used to design personalized bereavement interventions for Partners in Hospice Care (PHC)	Adjustment to daily life and role reconstruction; Modularity and dynamic organization	Not reported
Holtslander et al. (35)	Caregivers aged 60 or older whose spouse has died of advanced cancer	23	Bereavement after the death of a spouse from advanced cancer, with prior caregiving experience and access to palliative care services.	Expressive writing	2 weeks	Developed the “Finding Balance” Intervention and led the Inventory of Daily Widowed Life (IDWL) evaluation	Emotional expression and coping with loss; Adjustment to daily life and role reconstruction	Hope level, grief reactions, coping with bereavement, coping balance
Chow et al. (36)	Widowed individuals aged ≥60 with an ICG score >22	125	Within 2 years of the spouse’s death and exhibiting significant symptoms of grief	Group psychosocial intervention	8 weeks	DPM serves as the theoretical framework for dual-process bereavement group intervention-Chinese (DPBGI-C)	Emotional expression and coping with loss; Adjustment to daily life and role Reconstruction; Cultural specificity and Localization practices; Modularity and dynamic organization	Sadness, anxiety, depression, emotional loneliness, social loneliness, social support
Patterson et al. (37)	Adolescents and young adults aged 12–25 who have lost a parent or sibling to cancer	109	Grief support for adolescents and young adults who have lost a parent or sibling to cancer	Group psychological and social interventions	3 days and 2 nights	DPM was combined with frameworks such as meaning-making and continuing bonds to develop the Good Grief program	Emotional expression and coping with Loss; Meaning reconstruction and sustained connection	Intensity of grief, meaning-making, trauma coping ability, unmet needs, program acceptance, and appropriateness
Supiano et al. (38)	Family caregivers of people with dementia who are at risk for complicated grief	25	End-of-life care for patients with dementia who have a life expectancy of ≤6 months and are residing in a nursing home	Group psychotherapy	10 weeks	DPM and the theory of Meaning Reconstruction are used together to understand changes in complicated grief	Emotional expression and coping with loss; Adjustment to daily life and role reconstruction; Meaning reconstruction and sustained connection	Pre-loss grief; death preparedness; meaning-making; depression; anxiety; caregiving or life stress.
Debrot et al. (40)	People who have experienced the loss of a close relationship, including those who have lost a loved one to death or who have experienced a breakup with a romantic partner or spouse	62	Death, bereavement, or separation from a close partner lasting more than 6 months	Digital self-help intervention	10 online courses	Develop the LIVIA 2.0 online intervention	Emotional expression and coping with loss; Adjustment to daily life and role reconstruction; Modularity and dynamic organization	Symptoms of sadness, depression, well-being, anxiety, loneliness, satisfaction
Yu et al. (39)	Those Who Have Lost Loved Ones to COVID-19 in Wuhan	45	Traumatic bereavement caused by the COVID-19 pandemic	Internet-based + combined group and individual interventions	10 months	Designing a BTP framework	Adjustment to daily life and role reconstruction; Modularity and dynamic organization	Complicated grief
Jin et al. (41)	Chinese adulthood bereaved individuals with symptoms of prolonged grief	38	Those who have been bereaved for more than 6 months and exhibit symptoms of prolonged grief	WeChat mini-program	6 weeks	Developed the “Healing Grief” online self-help intervention	Emotional expression and coping with loss; Adjustment to daily life and role reconstruction; Meaning reconstruction and sustained connection; Modularity and dynamic organization	Symptoms, post-traumatic stress symptoms, anxiety, depression, post-traumatic growth, user experience
Phillips et al. (42)	Parents who have lost children to cancer	23	Support Settings for Bereaved Parents Between 6 Months and 5 Years After the Death of a Child from Cancer	Online group expressive therapy	6 weeks	DPM and the Meaning Reconstruction model jointly guide Storytelling Through Music (STM) interventions	Emotional expression and coping with loss; Meaning reconstruction and sustained connection	Prolonged grief, loneliness, anxiety, depression, self-compassion, ongoing connection
Zhou et al. (43)	Adults who have lost family members in an earthquake and are experiencing significant psychological distress	60	Adults who lost loved ones in the earthquake were recruited approximately 4–5 months after the earthquake and were experiencing psychological distress.	Group bereavement intervention	6 weeks	To develop a Confucian-based dual-process bereavement group intervention (C-DPBGI) adapted to Confucian culture	Emotional expression and coping with loss; Adjustment to daily life and role reconstruction; Meaning reconstruction and sustained connection; Cultural specificity and localization practices; Modularity and dynamic organization	Post-traumatic stress symptoms, general mental health distress
Dababneh et al. (44)	Adult refugees who have lost a loved one and are experiencing prolonged grief while in the transitional phase of their asylum application	116	Bereaved refugees who have lost a close relative at least 1 year ago and reside in Jordan	Online thematic writing intervention	1 week	To design thematic writing tasks	Emotional expression and coping with loss; Modularity and dynamic organization	Prolonged grief, positive mental health, depression, anxiety
Zhou et al. (45)	Chinese bereaved individuals with prolonged grief symptoms	133	Have lost a parent, spouse, or child ≥4 months ago and are experiencing grief	Network-based Modular Interventions	10 weeks	As the core theoretical framework of Grief Modular Group Therapy (GMGT)	Emotional expression and coping with loss; Adjustment to daily life and role reconstruction; Meaning reconstruction and sustained connection; Cultural specificity and localization practices; Modularity and dynamic organization	Prolonged symptoms of grief, symptoms of post-traumatic stress

### 3.3 Conceptual framework

To improve readability and summarize the main trends identified in the included studies, this study developed a conceptual framework for applying DPM in end-of-life and bereavement interventions, based on Table 2 and the included literature (Figure 2). This figure provides an integrated overview of the DPM structure, application methods, target populations, research contexts, and key outcome measures.

**Figure 2:**
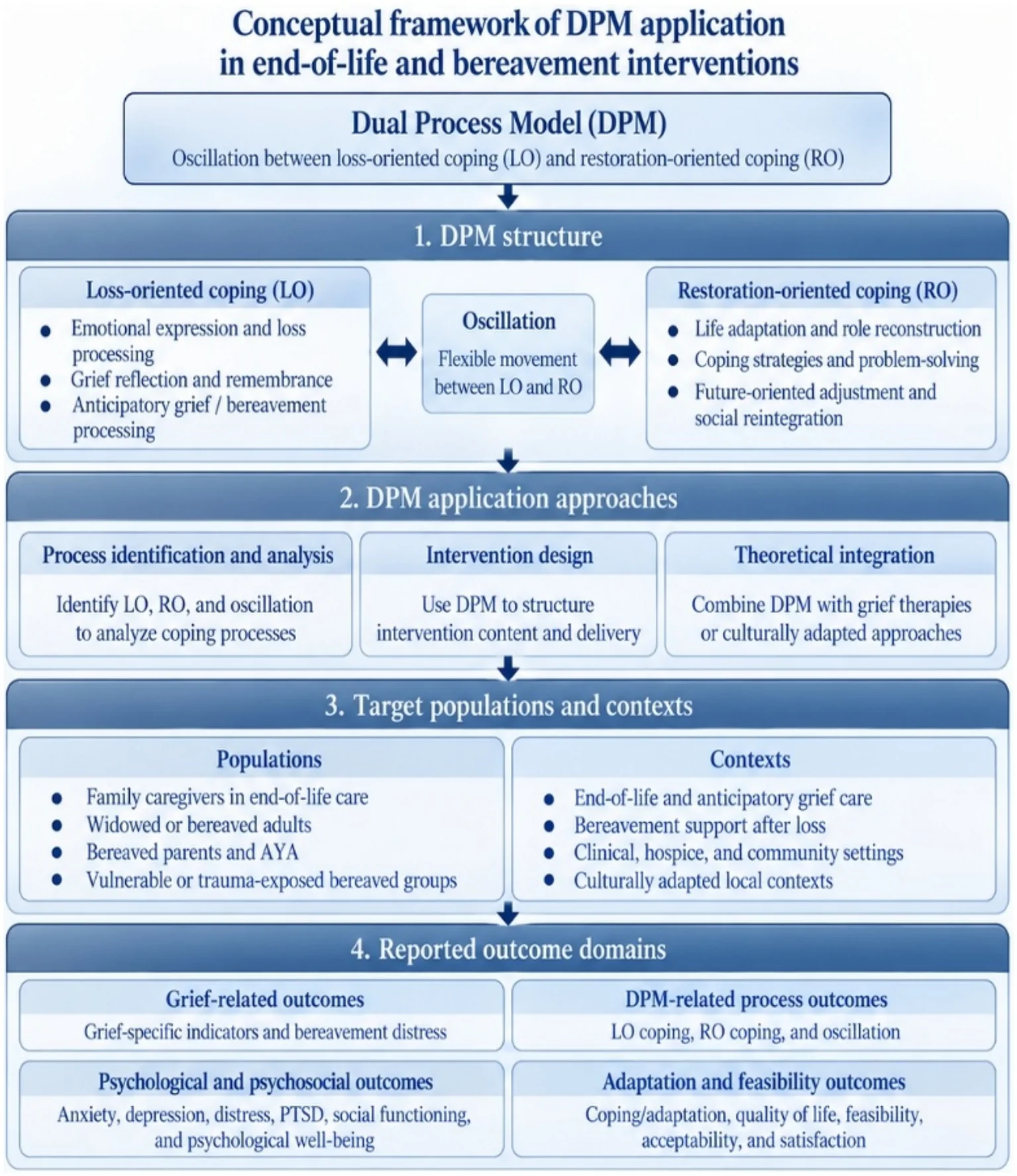
Conceptual framework of DPM application in end-of-life and bereavement interventions.

### 3.4 Target population and research contexts of DPM-based interventions

In terms of participant characteristics, the bereaved population primarily consisted of individuals who had lost a spouse or partner (*n* = 4, 23.5%) (29–31, 36); there were a total of 6 studies (35.3%) focusing on complicated grief, prolonged grief, and individuals at high risk for these conditions (32, 38, 40, 41, 44, 45). By kinship role, these included older bereaved individuals (*n* = 6, 35.3%) (29–32, 35, 36), adolescents and young adults (AYA) who had lost a loved one to cancer (*n* = 1, 5.9%) (37), and parents who had lost a child (*n* = 1, 5.9%) (42). There were a total of 4 studies (23.5%) related to caregivers, primarily involving support for bereavement or anticipatory grief in palliative care, end-of-life, or dementia care settings (33–35, 38). In addition, there were three studies (17.6%) focusing on vulnerable or trauma-exposed populations, involving individuals who had lost loved ones to COVID-19, survivors who had lost loved ones in an earthquake, and refugees who had lost loved ones and were in the transitional phase of their asylum applications (39, 43, 44). In terms of research settings, a total of 6 studies (35.3%) were conducted within networks specializing in end-of-life care, oncology, or palliative care (33–35, 37, 38, 42); Three studies (17.6%) were based on public health crises and disaster-related trauma (39, 43, 44); and five studies (29.4%) were conducted within the Chinese cultural context and included cultural adaptation (36, 39, 41, 43, 45).

### 3.5 Applications of DPM

The DPM applications included in the study can be divided into three categories. (1) Process identification and analysis, in which DPM is used as a framework for identifying LO/RO, and oscillatory processes, or for analyzing or evaluating them (*n* = 5, 29.4%) (29, 31, 33, 35, 44). Among these, some studies included LO, RO, and oscillation as process-based outcome measures (29, 31, 35, 44), while one study used DPM to analyze bereavement coping content in an online support group (33). (2) Intervention design, using DPM as a structured framework for organizing intervention content and delivery logic (*n* = 11, 64.7%) (29–31, 34–36, 39, 40, 43–45). These studies primarily focus on alternating, combining, modularizing, or individualizing the content of LO and RO, including fixed alternating curricula, flexible delivery based on individual needs, elective options, and modular group therapy. (3) Theoretical integration, combining DPM with treatments for complicated grief, meaning reconstruction, ongoing connection, cognitive-behavioral techniques, or culturally adapted frameworks in intervention design (*n* = 6, 35.3%) (32, 37, 38, 42, 43, 45). Among these, some studies incorporated elements such as role reconstruction, continuity of values, family responsibilities, or ritual practices into the RO content to expand the application of DPM in specific cultural or clinical contexts.

### 3.6 Types of DPM-based interventions

The types of interventions included in the study can be divided into three categories. (1) Face-to-face interventions. A total of 8 studies (29–32, 36–38, 43), primarily consisting of structured group therapy, individual psychotherapy, and camp activities. These interventions typically require on-site organization and guidance by professionals and involve face-to-face interactions in a designated setting. (2) Digital and self-directed interventions. There were a total of four studies (35, 40, 41, 44), which primarily utilized web platforms, mini-programs, and structured writing. These interventions transform DPM-related content into modular LO/RO-related content that participants can complete online or independently, providing them with support in coping with loss and adjusting to life changes. (3) Social Media Support and Blended Interventions. A total of five studies (33, 34, 39, 42, 45) were identified. These primarily included social media support, multi-modal follow-up, and blended group and individual interventions. Through platforms such as Facebook, WeChat, video conferencing, telephone, and email, these interventions integrated peer sharing, written feedback, narrative exposure, and ongoing follow-up into the intervention process.

### 3.7 Characteristics of DPM-based intervention content

The intervention content included in the study can be categorized into five types.

1. Emotional Expression and Coping with Loss, which primarily corresponds to LO. Intervention content is designed around the death event, the relationship with the deceased, and emotional reactions following bereavement, and includes grief education, group discussions, emotional expression, expressive writing, narrative exposure, guided imagery, memory processing, and symbolic expressions related to the deceased (29–33, 35–38, 40–45). This type of content is primarily used to help participants cope with grief, loneliness, guilt, longing, and painful memories related to death; it is the main manifestation of the loss-oriented approach in DPM interventions.
2. Adjustment to daily life and role reconstruction, which primarily corresponds to RO. Most studies focus their interventions on coping with the pressures of daily life following bereavement, covering topics such as self-care, health management, social connections, handling family matters, coping with caregiving stress, planning for the future, and adapting to new roles (29–31, 34–36, 38–41, 43–45). This section primarily addresses the needs for rebuilding one’s life and restoring social functioning after the loss of a loved one, reflecting the practical application of DPM’s restoration-oriented approach.
3. Meaning Reconstruction and Sustained Connection: Exhibits characteristics of both LO and RO. Some studies have helped participants reinterpret the relationship between the deceased and their own lives through methods such as reminiscence therapy, storytelling, music composition, active memory recognition, the creation of commemorative works, and the perpetuation of values (32, 37, 38, 41–43, 45). This type of content involves both reflecting on and synthesizing experiences of loss and reconstructing the meaning of future life and personal value; as such, it exhibits characteristics that straddle both LO and RO.
4. Cultural Specificity and Localization Practices: As cultural vehicles for expressing DPM. Studies conducted within the Chinese cultural context have incorporated concepts such as health preservation, traditional Chinese dietary practices, Qingming tomb-sweeping rituals, animal release ceremonies, and the Confucian concept of responsibility into intervention modules (36, 43, 45). On the one hand, these elements provide localized avenues for expressing grief; on the other hand, they embed the restoration of daily life, family responsibilities, and the reconstruction of social roles within cultural practices.
5. Modularity and dynamic organization: This corresponds to the oscillating transition approach in DPM. In the included studies, intervention content was organized through alternating LO/RO courses, collaborative tasks, menu-based modules, or individualized choices (29–31, 34, 36, 39–41, 43–45). Among these, structured group interventions often employ phased or alternating arrangements, while digital and self-help interventions primarily utilize module selection and task combinations to provide participants with practical pathways for dynamic transitions between coping with loss and resuming daily life.

### 3.8 Outcome measures for DPM-based intervention

1. Grief-specific outcomes. The outcome measures included in the study focused primarily on the assessment of grief-related symptoms. Grief-specific outcomes were reported most frequently (*n* = 12, 70.6%) (30, 32, 35–42, 44, 45), including overall grief reaction, complicated grief, and prolonged grief; commonly used instruments included the Complicated Grief Scale and the PGD Scale. Among these, comorbid emotional and traumatic symptoms (*n* = 11, 64.7%) (30, 32, 33, 36, 38, 40–45) primarily involved depression, anxiety, post-traumatic stress symptoms, and general psychological distress. Commonly used assessment tools included the Hospital Anxiety and Depression Scale, the Impact of Event Scale, and post-traumatic stress-related scales.
2. DPM-related process-oriented outcomes. Compared with grief-specific outcomes, fewer studies (*n* = 7, 41.2%) focused on DPM-related process variables (29, 31, 35, 37, 38, 40, 42). These primarily include LO/RO coping, the oscillation process, meaning-making, and persistent attachment. However, despite its central role in DPM theory, oscillation remains insufficiently examined regarding its measurement and operationalization. Existing research has primarily inferred the oscillation process indirectly through changes in LO- and RO-related indicators or through the integration of LO and RO components within interventions. However, standardized measurement tools that can directly assess individuals’ dynamic transitions between the two coping orientations remain lacking. Some studies have evaluated LO, RO, and oscillation-related coping responses, providing valuable insights into the DPM process. However, these assessments have primarily focused on changes in LO and RO coping orientations, without further examining the dynamic transition patterns underlying the oscillation process (29, 31, 35).
3. Psychosocial functioning and positive adaptation outcomes. Indicators related to psychosocial adaptation were reported in 8 studies (47.1%) (30, 32, 35, 36, 40–42, 44), including feelings of loneliness, social support, levels of hope, activities of daily living, self-care abilities, and post-traumatic growth. These indicators are often used to reflect changes in bereaved individuals regarding the reestablishment of daily life, the restoration of social functioning, and positive psychological adaptation.
4. Feasibility and acceptability outcomes. A total of 4 studies (23.5%) used formal satisfaction or acceptability questionnaires to report outcomes related to intervention implementation (29, 37, 40, 41), primarily addressing satisfaction, acceptability, usability, the participant experience, and the extent to which the intervention met the needs of the target population.

## 4 Discussion

### 4.1 Differences in support needs among expanding populations for DPM

The results of this study indicate that the target population for DPM has gradually expanded from the initially more concentrated group of individuals who have lost a spouse or partner (29–31) to include those experiencing illness-related bereavement, complicated or prolonged grief, and bereavement resulting from crisis situations such as disasters, pandemics, and refugee crises (32, 35–45). This finding indicates that DPM has been applied to a variety of bereaved populations and contexts, but support needs vary across different groups. Previous research has shown that factors such as accidental or unnatural death, the loss of a child or spouse, and insufficient social support may all increase the risk of prolonged grief (9). Therefore, the application of DPM should not only focus on expanding the target population but also on paying closer attention to the differences in risk stratification and support needs across groups. The public health bereavement support model proposed by Aoun et al. emphasizes that the support needs of bereaved individuals vary across different levels, and that universal support, selective support, and specialized interventions should be tailored according to risk levels and support needs (46). Within this tiered system, DPM can further provide a basis for tailoring interventions. For individuals mainly experiencing loss-related stressors, such as grief, longing, guilt, or traumatic memories, loss-oriented support should be prioritized. For those mainly facing restoration-oriented stressors, such as disrupted routines, role changes, weakened social connections, or practical difficulties, restoration-oriented support should be strengthened. Included in this study, individualized bereavement interventions in primary care and pre-bereavement group therapy have tentatively demonstrated the approach of dynamically adjusting the focus of support based on individual needs (34, 38). However, most studies have not sufficiently integrated risk screening, needs assessment, and the matching of LO or RO content into a clear intervention pathway. Future research should further clarify the risk stratification and support needs of different bereaved populations within the DPM framework, and optimize the selection of intervention recipients and the allocation of support.

### 4.2 Insufficient application of DPM during the end-of-life phase and the bereavement transition period

This study found that existing DPM interventions are primarily focused on the bereavement adjustment phase following death (29–33, 35–37, 39–45), with only a few studies extending the application of DPM to the end-of-life phase or covering the transition from palliative care to post-death bereavement support (34, 38). These findings indicate that the application of DPM in grief support primarily focuses on post-death bereavement adjustment, with relatively less attention paid to the end-of-life care phase and the transitional periods before and after death. Recent studies have shown that family caregivers’ grief experiences may begin even before the patient’s death and are associated with the burden of care, social support, and post-death grief reactions (21, 47). At the same time, research on palliative care caregivers indicates that existing interventions primarily focus on outcomes such as caregivers’ quality of life, anxiety, and care burden. However, most of these interventions are still in the early stages of development, and their efficacy, implementation, and models of continuous support before and after death require further validation (48). During the end-of-life stage, family caregivers experience both loss-oriented stress associated with disease progression and changes in their relationship with the patient, as well as restoration-oriented stress arising from caregiving responsibilities, role adjustments, and future life planning. These findings suggest that DPM is applicable not only after bereavement but also during the pre-loss caregiving period, providing targeted support by identifying caregivers’ evolving needs. At the end-of-life stage, DPM provides a theoretical framework for palliative care teams to identify family caregivers’ diverse needs as they experience anticipated loss. Following the patient’s death, DPM-informed grief interventions can be implemented to facilitate a continuous transition from end-of-life caregiving to bereavement adjustment. Therefore, future research should further explore DPM-based models of continuous care spanning the period before and after death, to avoid treating palliative care and bereavement support as two separate stages of care.

### 4.3 The dual-task orientation of the intervention content model: the process of coping still needs to be strengthened

This study found that the intervention content primarily consisted of two task types: LO and RO. The former focused on expressing grief, processing relationships with the deceased, and reconstructing meaning, while the latter focused on self-care, role adaptation, rebuilding one’s life, and social connection. This suggests that existing DPM interventions exhibit, to some extent, a dual task orientation corresponding to LO and RO approaches. However, the core of DPM is not merely to provide both types of support simultaneously, but rather to emphasize the individual’s dynamic oscillation between loss-oriented and recovery-oriented perspectives (17). Research further suggests that LO and RO stressors may serve as important process factors influencing grief outcomes, and that flexible adjustment of coping strategies is associated with long-term bereavement adaptation (49, 50). Some studies have organized LO and RO content through alternating courses, modular tasks, or individualized delivery methods (29–31, 34, 40, 44), but they provide insufficient explanation of how oscillation is guided, tracked, and on what is feedback provided during the intervention process. Different intervention delivery methods may influence the realization of the oscillation process. For example, face-to-face interventions allow therapists to engage in ongoing interaction with participants, promptly identify changes in an individual’s grief response, and adjust the support provided through LO and RO based on the participant’s current needs, thereby creating the conditions for oscillation. In contrast, digital interventions can enhance flexibility and promote self-directed participation, allowing individuals to select content based on their own conditions. However, some digital formats currently still rely primarily on preset modules and self-guided tasks, and their ability to support emotional state recognition, real-time feedback, and module transitions still requires further exploration. Research by Carey also suggests that theory needs to be further translated into specific mechanisms and intervention techniques capable of bringing about change (51). Therefore, future DPM-informed interventions should extend beyond the integration of LO and RO components by incorporating strategies that facilitate, monitor, and provide feedback on the oscillation process, such as coping-switching exercises and dynamic diaries. These approaches may help operationalize oscillation as an observable and potentially modifiable process.

### 4.4 Lack of process-oriented measures in outcome assessments

The outcome assessments in the studies primarily focused on measures of grief intensity, complexity, or prolonged grief. These measures help assess the impact of interventions on symptom relief and psychosocial status, aligning with the clinical needs for PGD identification and mental health assessment (39, 40). However, from the perspective of DPM theory, grief adaptation is not merely characterized by a reduction in symptoms, but rather is a continuous process of adjustment as the individual navigates the transition from coping with loss to resuming normal life (16). Previous studies have shown that process variables such as rumination, avoidance, and negative grief cognitions are closely associated with the persistence of complicated or protracted grief and the effectiveness of interventions (52, 53); RO stress may also influence grief and depression outcomes among bereaved individuals (54). Current research relies primarily on symptom-focused measures, which may not adequately capture the dynamic oscillation between LO- and RO-oriented coping over time, as proposed in DPM theory. This limitation may reflect a potential mismatch between traditional psychometric instruments and the theoretical assumptions underlying DPM. Existing studies often use scales related to depression, anxiety, PTSD, and grief as primary outcome measures. These tools are primarily used to assess an individual’s symptom levels, psychological distress, or the severity of grief-related reactions. However, current measurement tools still focus mainly on symptom assessment and pay relatively limited attention to the dynamic changes occurring during the grief adaptation process (55). DPM emphasizes the dynamic transitions in coping patterns over time throughout the grief adaptation process (16). Although static symptom indicators can reflect changes in outcomes following an intervention, they may not adequately capture how individuals adjust between loss-oriented coping and restoration-oriented coping, or how this oscillation process contributes to adaptive grief adaptation. Therefore, future DPM-informed interventions should go beyond integrating LO and RO components by incorporating strategies that facilitate, monitor, and provide feedback on the oscillation process, such as coping-switching exercises and dynamic diaries. These approaches may help operationalize oscillation as an observable and potentially modifiable process.

### 4.5 Digital implementation and cultural adaptation still face challenges in practical application

This study found that web platforms, WeChat mini-programs, online support groups, expressive writing, and video conferencing have been used in DPM interventions (33–35, 39–42), indicating that digital methods provide more flexible avenues for implementing bereavement support. Findings by Finucane et al. on online bereavement support indicate that online interventions are generally feasible and acceptable, and may improve grief intensity, stress-related outcomes, and depressive symptoms (56). Another study on web-based grief intervention highlighted that such interventions can improve grief, anxiety, depression, and PTSD symptoms immediately following the intervention, but the long-term maintenance effects for some outcomes remain limited (57). Nevertheless, from the perspective of DPM theory, the value of digital interventions extends beyond providing content related to LO and RO to supporting individuals in dynamic adjustment between the two coping orientations. Some digital interventions primarily take the form of pre-set courses, self-directed writing, or online communication. These approaches may provide limited ongoing interaction, individualized feedback, and monitoring of changes during the intervention (40, 41, 44). Furthermore, the LO process involves more than information acquisition. It includes emotional engagement with the loss, reinterpreting the relationship with the deceased, and meaning-making. These processes often require sustained emotional involvement, individualized feedback, and timely responses to emotional changes. In comparison, some digital interventions have attempted to support these processes through structured writing, online modules, and guided tasks. They still face challenges in capturing subtle changes in an individual’s emotions, providing relational support, and dynamically adjusting intervention content based on grief reactions. Therefore, future DPM-based digital interventions need to enhance support for LO emotional processing and the dynamic regulatory processes between LO and RO, while maintaining accessibility and flexibility.

In addition to changes in delivery methods, the cultural adaptation of DPM interventions across different cultural contexts is another key factor influencing their application. In terms of cultural adaptation, studies conducted within the Chinese cultural context have incorporated elements such as tomb-sweeping, releasing living creatures, the Confucian concept of responsibility, and the continuation of familial bonds into DPM interventions (36, 43, 45). These elements are related to maintaining relationships with the deceased, assuming family responsibilities, and reconstructing the meaning of life. Research on cultural adaptation emphasizes that the cultural adaptation of psychosocial interventions should focus on the content of the intervention, the context of implementation, the participation of the target population, and the competence of the implementers—rather than merely incorporating local elements at a superficial level (58). Future research should not be limited to simply increasing the amount of culturally specific content; it should also evaluate the acceptability and effectiveness of culturally adapted interventions, thereby promoting the application of DPM interventions in diverse cultural and nursing care settings.

### 4.6 Limitations

This systematic review has certain limitations. First, this study included only Chinese- and English-language literature, potentially omitting DPM-related intervention studies published in other languages. Second, since the DPM can be applied to different bereaved populations and contexts, and to avoid omitting relevant studies by limiting the search to specific populations, this study did not use specific bereaved populations as a search criterion during the literature search phase; instead, the study population was determined based on the inclusion and exclusion criteria during the literature screening phase. Finally, this study did not formally assess the methodological quality of the included studies; therefore, it is not possible to further compare the methodological quality of different studies or the reliability of their reports on intervention outcomes. Future high-quality studies are needed to further evaluate the effectiveness and implementation of DPM-informed interventions.

## 5 Conclusion

This study systematically reviewed the current status of DPM’s application in end-of-life and bereavement interventions. The results indicate that DPM provides a theoretical perspective for addressing the dual needs of coping with loss and resuming normal life in end-of-life and bereavement interventions. Existing research suggests that DPM-based interventions hold some potential for application in end-of-life and bereavement support, but their use remains in the exploratory and translational stages. Current research primarily focuses on bereavement adjustment following a death, with insufficient attention paid to anticipatory grief among family members of terminally ill patients and to continuous support before and after death. At the same time, the oscillation process in DPM remains insufficiently operationalized and measured, and outcome assessments have largely focused on indicators such as grief, depression, anxiety, and post-traumatic stress, with relatively insufficient attention paid to process-oriented indicators such as life reconstruction, dynamic regulation, and oscillation between LO and RO. Future research should further clarify the specific role of DPM in the development, implementation, and evaluation of interventions within the contexts of palliative care, community care, and continuity of care. By integrating digital delivery with cultural adaptation, researchers should establish a continuous grief support pathway that spans from the end-of-life stage through the bereavement period.

## Data Availability

The original contributions presented in the study are included in the article/supplementary material, further inquiries can be directed to the corresponding authors.

## References

[1] Bray F Laversanne M Sung H Ferlay J Siegel RL Soerjomataram I . Global cancer statistics 2022: GLOBOCAN estimates of incidence and mortality worldwide for 36 cancers in 185 countries. CA Cancer J Clin. (2024) 74:229–63. doi: 10.3322/caac.21834, 38572751

[2] World Health Organization. Global Health estimates 2021: Deaths by Cause, Age, Sex, by Country and by Region, 2000–2021. In: Geneva: World Health Organization (2024).

[3] Sallnow L Smith R Ahmedzai SH Bhadelia A Chamberlain C Cong Y . Report of the lancet commission on the value of death: bringing death back into life. Lancet. (2022) 399:837–84. doi: 10.1016/s0140-6736(21)02314-x, 35114146 PMC8803389

[4] GBD 2023 Causes of Death Collaborators. Global burden of 292 causes of death in 204 countries and territories and 660 subnational locations, 1990-2023: a systematic analysis for the Global Burden of Disease Study 2023 [J]. Lancet (2025) 406: 1811–1872. doi: 10.1016/s0140-6736(25)01917-8 PMC12535838 41092928

[5] Lichtenthal WG Roberts KE Donovan LA Breen LJ Aoun SM Connor SR . Investing in bereavement care as a public health priority. Lancet Public Health. (2024) 9:e270–4. doi: 10.1016/s2468-2667(24)00030-6, 38492580 PMC11110717

[6] Coelho A Albuquerque S Neto DD. Bereavement support guidelines for caregivers in palliative care: a scoping review. Front Psychol. (2025) 16:1541783. doi: 10.3389/fpsyg.2025.1541783, 40260008 PMC12010901

[7] Singer J Roberts KE McLean E Fadalla C Coats T Rogers M . An examination and proposed definitions of family members' grief prior to the death of individuals with a life-limiting illness: a systematic review. Palliat Med. (2022) 36:581–608. doi: 10.1177/02692163221074540, 35196915 PMC10098140

[8] Killikelly C Smith KV Zhou N Prigerson HG O'Connor M-F Kokou-Kpolou CK . Prolonged grief disorder. Lancet. (2025) 405:1621–32. doi: 10.1016/s0140-6736(25)00354-x, 40254022

[9] Buur C Zachariae R Komischke-Konnerup KB Marello MM Schierff LH O'Connor M. Risk factors for prolonged grief symptoms: a systematic review and meta-analysis. Clin Psychol Rev. (2024) 107:102375. doi: 10.1016/j.cpr.2023.102375, 38181586

[10] Comtesse H Smid GE Rummel AM Spreeuwenberg P Lundorff M Dückers MLA. Cross-national analysis of the prevalence of prolonged grief disorder. J Affect Disord. (2024) 350:359–65. doi: 10.1016/j.jad.2024.01.094, 38220101

[11] Eisma MC. Prolonged grief disorder in ICD-11 and DSM-5-TR: challenges and controversies. Aust N Z J Psychiatry. (2023) 57:944–51. doi: 10.1177/00048674231154206, 36748103 PMC10291380

[12] Prigerson HG Shear MK Reynolds CF 3rd. Prolonged grief disorder diagnostic criteria-helping those with maladaptive grief responses. JAMA Psychiatry. (2022) 79:277–8. doi: 10.1001/jamapsychiatry.2021.4201, 35107569

[13] Bartley N Rodriguez Grieve L Cooper C Kirsten L Wilson C Sajish B . A systematic review of international bereavement models of care and implementation barriers and facilitators. Omega (Westport). (2025):302228251315502. doi: 10.1177/00302228251315502, 39836068 PMC13646394

[14] Patinadan PV Tan-Ho G Choo PY Ho AHY. Resolving anticipatory grief and enhancing dignity at the end-of life: a systematic review of palliative interventions. Death Stud. (2022) 46:337–50. doi: 10.1080/07481187.2020.1728426, 32079501

[15] Schwalbach T Riguzzi M Kohler M Naef R. Family bereavement support interventions in specialist adult palliative care: a rapid mixed-methods systematic review. J Adv Nurs. (2026) 82:4682–713. doi: 10.1111/jan.70193, 40913300 PMC13069219

[16] Stroebe M Schut H. The dual process model of coping with bereavement: rationale and description. Death Stud. (1999) 23:197–224. 10.1080/074811899201046 10848151

[17] Stroebe M Schut H. The dual process model of coping with bereavement: a decade on. Omega (Westport). (2010) 61:273–89. doi: 10.2190/OM.61.4.b, 21058610

[18] Maciejewski PK Zhang B Block SD Prigerson HG. An empirical examination of the stage theory of grief. JAMA. (2007) 297:716–23. doi: 10.1001/jama.297.7.716, 17312291

[19] Neimeyer RA. Meaning reconstruction in bereavement: development of a research program. Death Stud. (2019) 43:79–91. doi: 10.1080/07481187.2018.1456620, 30907718

[20] Wortman CB Silver RC. The myths of coping with loss. J Consult Clin Psychol. (1989) 57:349–57. 10.1037//0022-006x.57.3.349 2661609

[21] Li C Tang N Yang L Zeng Q Yu T Pu X . Effect of caregiver burden on anticipatory grief among caregivers of elderly cancer patients: chain mediation role of family functioning and resilience. Front Psychol. (2022) 13:1020517. doi: 10.3389/fpsyg.2022.1020517, 36704702 PMC9872027

[22] Fiore J. A systematic review of the dual process model of coping with bereavement (1999-2016). Omega (Westport). (2021) 84:414–58. doi: 10.1177/0030222819893139, 31829782

[23] Larsen LH Hybholt L O'Connor M. Lived experience and the dual process model of coping with bereavement: a participatory research study. Death Stud. (2025) 49:743–54. doi: 10.1080/07481187.2024.2355244, 38822451

[24] Bennett KM Gibbons K Mackenzie-Smith S. Loss and restoration in later life: an examination of dual process model of coping with bereavement. Omega (Westport). (2010) 61:315–32. doi: 10.2190/OM.61.4.d, 21058612

[25] Skivington K Matthews L Simpson SA Craig P Baird J Blazeby JM . A new framework for developing and evaluating complex interventions: update of Medical Research Council guidance. Int J Nurs Stud. (2024) 154:104705. doi: 10.1016/j.ijnurstu.2024.104705, 38564982

[26] Munn Z Peters MDJ Stern C Tufanaru C McArthur A Aromataris E. Systematic review or scoping review? Guidance for authors when choosing between a systematic or scoping review approach. BMC Med Res Methodol. (2018) 18:143. doi: 10.1186/s12874-018-0611-x, 30453902 PMC6245623

[27] Peters MDJ Marnie C Tricco AC Pollock D Munn Z Alexander L . Updated methodological guidance for the conduct of scoping reviews. JBI Evid Implement. (2021) 19:3–10. doi: 10.1097/xeb.0000000000000277 33570328

[28] Tricco AC Lillie E Zarin W O'Brien KK Colquhoun H Levac D . PRISMA extension for scoping reviews (PRISMA-ScR): checklist and explanation. Ann Intern Med. (2018) 169:467–73. doi: 10.7326/m18-0850, 30178033

[29] Lund D Caserta M Utz R de Vries B. Experiences and early coping of bereaved spouses/partners in an intervention based on the dual process model (dpm). Omega (Westport). (2010) 61:291–313. doi: 10.2190/OM.61.4.c, 21058611 PMC3056082

[30] Utz RL Caserta M Lund D. An evaluation of the living after loss intervention - part 1: do bereavement-related outcomes vary by the content of support groups? Grief Matters. (2013) 16:32–5. Available online at: https://pubmed.ncbi.nlm.nih.gov/24976788/PMC4204648 24976788

[31] Caserta M Utz R Lund D Swenson KL de Vries B. Coping processes among bereaved spouses. Death Stud. (2014) 38:145–55. doi: 10.1080/07481187.2012.738767, 24524542 PMC3929225

[32] Shear MK Wang Y Skritskaya N Duan N Mauro C Ghesquiere A. Treatment of complicated grief in elderly persons: a randomized clinical trial. JAMA Psychiatry. (2014) 71:1287–95. doi: 10.1001/jamapsychiatry.2014.1242, 25250737 PMC5705174

[33] Wittenberg-Lyles E Washington K Oliver DP Shaunfield S Gage LA Mooney M . "it is the 'starting over' part that is so hard": using an online group to support hospice bereavement. Palliat Support Care. (2015) 13:351–7. doi: 10.1017/s1478951513001235, 24559689 PMC4143489

[34] Caserta MS Lund DA Utz RL Tabler JL. One size doesn't fit all-partners in hospice care, an individualized approach to bereavement intervention. Omega. (2016) 73:107–25. doi: 10.1177/0030222815575895, 27141124 PMC4851167

[35] Holtslander L Duggleby W Teucher U Cooper D Bally JMG Solar J . Developing and pilot-testing a finding balance intervention for older adult bereaved family caregivers: a randomized feasibility trial. Eur J Oncol Nurs. (2016) 21:66–74. doi: 10.1016/j.ejon.2016.01.003, 26952680

[36] Chow AYM Caserta M Lund D Suen MHP Xiu D Chan IKN . Dual-process bereavement group intervention (DPBGI) for widowed older adults. Gerontologist. (2019) 59:983–94. doi: 10.1093/geront/gny095, 30137473

[37] Patterson P McDonald FEJ Kelly-Dalgety E Lavorgna B Jones BL Sidis AE . Development and evaluation of the good grief program for young people bereaved by familial cancer. BMC Palliat Care. (2021) 20:64. doi: 10.1186/s12904-021-00752-z, 33926449 PMC8083089

[38] Supiano KP Andersen T Luptak M Beynon C Iacob E Levitt SE. Pre-loss group therapy for dementia family care partners at risk for complicated grief. Alzheimers Dement (N Y). (2021) 7:e12167. doi: 10.1002/trc2.12167, 34027022 PMC8116857

[39] Yu ZH Liang JJ Guo LP Jiang LR Wang JY Ke ML . Psychosocial intervention on the dual-process model for a group of COVID-19 bereaved individuals in Wuhan: a pilot study. Omega-J Death Dying. (2022) 89:701–17. doi: 10.1177/00302228221083067, 35341382 PMC8958304

[40] Debrot A Efinger L Kheyar M Pomini V Berthoud L. A French-language web-based intervention targeting prolonged grief symptoms in people who are bereaved and separated: randomized controlled trial. Jmir Formative Res. (2024) 8:e57294. doi: 10.2196/57294, 39412869 PMC11525088

[41] Jin WX Peng WJ Tang SQ. Acceptability and feasibility of an internet-based intervention for bereaved Chinese with prolonged grief: a mixed methods study. Eur J Psychotraumatol. (2025) 16:2484872. doi: 10.1080/20008066.2025.2484872, 40226897 PMC11998308

[42] Phillips CS Morris SE Rodriguez E Woods H Hebdon M Choi E . Storytelling through music with parents whose children have died from cancer: a randomized controlled feasibility trial. Psycho-Oncology. (2025) 34:e70143. doi: 10.1002/pon.70143, 40195024

[43] Zhou YP Liu L Wen XC Gao ZC Li YN Han SY . Confucian-based dual process bereavement group intervention (C-DPBGI) for bereaved survivors of the earthquake. Psychol Trauma. (2025) 18:1135–44. doi: 10.1037/tra0002018, 40758267

[44] Dababneh N Margraf J Ayoub Momani F Precht L-M Brailovskaia J. The effectiveness of online thematic expressive writing on prolonged grief disorder, anxiety, depression, and positive mental health among refugees in the transition stage of asylum seeking. J Immigr Minor Health. (2026) 28:360–8. doi: 10.1007/s10903-025-01778-8, 40938342 PMC13083514

[45] Zhou NN Tang SQ Zhou JQ Guo LP Lang JL Situ B . Grief modular group therapy: theoretical bases and pilot study of a new bereavement intervention programme in China. Eur J Psychotraumatol. (2026) 17:2651656. doi: 10.1080/20008066.2026.2651656, 41979437 PMC13081348

[46] Aoun SM Breen LJ Howting DA Rumbold B McNamara B Hegney D. Who needs bereavement support? A population based survey of bereavement risk and support need. PLoS One. (2015) 10:e0121101. doi: 10.1371/journal.pone.0121101, 25811912 PMC4374848

[47] Axelsson L Alvariza A Holm M Årestedt K. Intensity of Predeath grief and Postdeath grief of family caregivers in palliative Care in Relation to preparedness for caregiving, caregiver burden, and social support. Palliat Med Rep. (2020) 1:191–200. doi: 10.1089/pmr.2020.0033, 34223476 PMC8241336

[48] Alshakhs S Park T McDarby M Reid MC Czaja S Adelman R . Interventions for family caregivers of patients receiving palliative/hospice Care at Home: a scoping review. J Palliat Med. (2024) 27:112–27. doi: 10.1089/jpm.2023.0160, 37582194 PMC10790551

[49] Huang SH Birk JL Bonanno GA. Looking back and moving forward: dimensions of coping flexibility divergently predict long-term bereavement outcomes. Anxiety Stress Coping. (2023) 36:275–90. doi: 10.1080/10615806.2022.2099545, 35852939 PMC9849482

[50] Palm S Doering BK Kubiak T Geschke K Fellgiebel A Wuttke A. Influence of loss- and restoration-oriented stressors on grief in times of COVID-19. Sci Rep. (2023) 13:19584. doi: 10.1038/s41598-023-46403-6, 37949946 PMC10638438

[51] Carey RN Connell LE Johnston M Rothman AJ de Bruin M Kelly MP . Behavior change techniques and their mechanisms of action: a synthesis of links described in published intervention literature. Ann Behav Med. (2019) 53:693–707. doi: 10.1093/abm/kay078, 30304386 PMC6636886

[52] Eisma MC Stroebe MS. Emotion regulatory strategies in complicated grief: a systematic review. Behav Ther. (2021) 52:234–49. doi: 10.1016/j.beth.2020.04.004, 33483120

[53] Lechner-Meichsner F Mauro C Skritskaya NA Shear MK. Change in avoidance and negative grief-related cognitions mediates treatment outcome in older adults with prolonged grief disorder. Psychother Res. (2022) 32:91–103. doi: 10.1080/10503307.2021.1909769, 33818302 PMC8490492

[54] Eisma MC de Lang TA Stroebe MS. Restoration-oriented stressors of bereavement. Anxiety Stress Coping. (2022) 35:339–53. doi: 10.1080/10615806.2021.1957849 34338103

[55] Janshen A Eisma MC. Bidirectional associations between prolonged grief symptoms and depressive, anxiety, and posttraumatic stress symptoms: a systematic review. J Trauma Stress. (2024) 37:825–36. doi: 10.1002/jts.23061, 38924632 PMC11629839

[56] Finucane A Canny A Mair APA Harrop E Selman LE Swash B . A rapid review of the evidence for online interventions for bereavement support. Palliat Med. (2025) 39:31–52. doi: 10.1177/02692163241285101, 39407434 PMC11673319

[57] Yao DY Qian F Tung TH Shi H Bi D. The effectiveness of web-based grief intervention for adults who lost a loved one: a systematic review and meta-analysis. BMC Palliat Care. (2025) 24:61. doi: 10.1186/s12904-025-01679-5, 40057748 PMC11889856

[58] Day S Laver K Jeon YH Radford K Low LF. Frameworks for cultural adaptation of psychosocial interventions: a systematic review with narrative synthesis. Dementia (London). (2023) 22:1921–49. doi: 10.1177/14713012231192360, 37515347 PMC10644683

